# Association between Income and the Hippocampus

**DOI:** 10.1371/journal.pone.0018712

**Published:** 2011-05-04

**Authors:** Jamie L. Hanson, Amitabh Chandra, Barbara L. Wolfe, Seth D. Pollak

**Affiliations:** 1 Department of Psychology, University of Wisconsin-Madison, Madison, Wisconsin, United States of America; 2 Waisman Center, University of Wisconsin-Madison, Madison, Wisconsin, United States of America; 3 Harvard Kennedy School of Government, Harvard University, Cambridge, Massachusetts, United States of America; 4 Departments of Economics, Population Health Sciences and Public Affairs, and Institute for Research on Poverty, University of Wisconsin-Madison, Madison, Wisconsin, United States of America; University of Michigan, United States of America

## Abstract

Facets of the post-natal environment including the type and complexity of environmental stimuli, the quality of parenting behaviors, and the amount and type of stress experienced by a child affects brain and behavioral functioning. Poverty is a type of pervasive experience that is likely to influence biobehavioral processes because children developing in such environments often encounter high levels of stress and reduced environmental stimulation. This study explores the association between socioeconomic status and the hippocampus, a brain region involved in learning and memory that is known to be affected by stress. We employ a voxel-based morphometry analytic framework with region of interest drawing for structural brain images acquired from participants across the socioeconomic spectrum (n = 317). Children from lower income backgrounds had lower hippocampal gray matter density, a measure of volume. This finding is discussed in terms of disparities in education and health that are observed across the socioeconomic spectrum.

## Introduction

A growing body of research, conducted mainly in rodents, has found that factors such as the complexity of stimuli present in the post-natal environment, the quality of parenting behaviors, and the amount of stress that occurs during the lifespan can affect neural, emotional and cognitive functioning (for review, see [1], [2]). These findings raise complex questions about how variations in the environment can shape neural development in humans [3]. In particular, an increasing interest is being paid to the effects of socioeconomic status and poverty on brain and behavior, since living in poverty is often characterized by heightened amounts of stress and reductions in environmental stimulation [4].

This study focuses on associations between household income and the hippocampus. The hippocampus is located in the medial temporal lobe of the brain. This region is known to be affected by stress and is tied to cognitive functions such as learning, memory, and behavioral regulation (for review, see [5]). It is difficult to quantify the many facets of an individual's environment; for this reason, we use income as a proxy for a multitude of factors including enriched cultural environment, better schools and neighborhoods, and access to stimulating materials in early childhood.

Non-human animal research has found environmental enrichment is related to greater dendritic branching and wider dendritic fields [6], [7], increased astrocyte number and size [8], and improved synaptic transmission [9] in portions of the hippocampus. Environmental enrichment, in addition, appears to bolster neurobiological resiliency. For example, enriched environments result in increases in neuronal precursor cells in portions of the hippocampus [10] and greater recovery after a lesion in the hippocampus [11]. Stress also exerts long-lasting negative effects on the hippocampus. For example, research has found prolonged maternal separation and brief handling impacts the hippocampus and affects stress regulation and memory ability later in life [12]. Similar effects have been noted in humans. These studies suggest that parental nurturance and environmental stimulation, including both resources such as the number of books in a child's home and parental time spend reading to a child, predict neurocognitive performance on tests related to the hippocampus such as long-term memory [13], [14].

Prior research has linked poverty with a myriad of deleterious outcomes from poor health to lower educational achievement [15], [16], [17], [18]. Yet little is currently understood about the neurobiological mechanisms leading to these socioeconomic disparities. We hypothesized that the morphometric properties of hippocampus would be related to gradients in income. We focus on this brain region both because of its known sensitivity to environmental stress and its role in core adaptive processes such as learning.

## Methods

### Subjects and MRI acquisition

Behavioral and MRI data were taken from the National Institutes of Health (NIH) MRI study of normal brain development (website: http://nihpd.crbs.ucsd.edu/nihpd/info/index.html and [19]). This public-access database was developed by the NIH to aid in understanding the course of normal brain-behavior development. The database consists of clinical, behavioral and neuroimaging metrics that were acquired at multiple research centers across the US from a large cohort of children and adolescents ages 4 to 18. To participate in the study, subjects had to meet criteria based on demographic, prenatal history, physical, behavioral/psychiatric, family history, and neurological exam cutoffs (Exclusion criteria are listed in Table 1; adapted from [20]). Families whose child met all inclusion and no exclusion criteria were invited to participate in neurological evaluation, neuropsychological testing, and structural MRI imaging, typically performed in one day. Written informed consent from the parents/guardians of all children was obtained in compliance with research standards for human research at Boston Children's Hospital, Cincinnati Children's Hospital, Philadelphia Children's Hospital, Washington University in St. Louis, the University of Texas Health Science Center in Houston, and the University of California in Los Angeles. Children ages 6 to 17, in addition, gave their written assent. These procedures were in accordance with the Helsinki Declaration. The Institutional Review Board at the University of Wisconsin-Madison also approved the analysis of this human subjects data.

**Table 1:** Exclusionary criteria (originally appeared in [20] © Cambridge Journals, reproduced with permission.)

Category	Specific criteria
Demographic	Children of parents with limited English proficiency. Adopted children excluded due to inadequate family histories.
Pregnancy, birth and perinatal history	Intra-uterine exposures to substances known or highly suspected to alter brain structure or function (certain medications, any illicit drug use, smoking >.5 pack per day or >2 alcoholic drinks per week during pregnancy); Hyperbilirubinemia requiring transfusion and0or phototherapy (>2 days); gestational age at birth of <37 weeks or >42 weeks; multiple birth; delivery by high forceps or vacuum extraction; infant resuscitation by chest compression or intubation; maternal metabolic conditions (e.g., phenylketonuria, diabetes); pre-eclampsia; serious obstetric complication; general anesthesia during pregnancy/delivery; C-section for maternal or infant distress
Physical/medical or growth	Current height or weight <3rd percentile or head circumference <3rd percentile by National Center for Health Statistics 2000 data (charts at http://www.cdc.gov/nchs/about/major/nhanes/growthcharts/charts.htm); history of significant medical or neurological disorder with CNS implications (e.g., seizure disorder, CNS infection, malignancy, diabetes, systemic rheumatologic illness, muscular dystrophy, migraine or cluster headaches, sickle cell anemia, etc.); history of closed head injury with loss of consciousness >30 min or with known diagnostic imaging study abnormalities; systemic malignancy requiring chemotherapy or CNS radiotherapy; hearing impairment requiring intervention; significant visual impairment requiring more than conventional glasses (strabismus, visual handicap); metal implants (braces, pins) if likely to pose safety or artifact issues for MRI; positive pregnancy test in subject.
Behavioral/psychiatric	Current or past treatment for language disorder (simple articulation disorders not exclusionary); lifetime history of Axis I psychiatric disorder (except for simple phobia, social phobia, adjustment disorder, oppositional defiant disorder, enuresis, encopresis, nicotine dependency); any CBCL subscale score ≥70; WASI IQ<70; Woodcock-Johnson Achievement Battery subtest score <70; current or past treatment for an Axis I psychiatric disorder.
Family history	History of inherited neurological disorder; history of mental retardation caused by non-traumatic events in any first-degree relative; one or more first degree relatives with lifetime history of Axis I psychiatric disorders; schizophrenia, bipolar affective disorder, psychotic disorder, alcohol or other drug dependence, obsessive compulsive disorder, Tourette's disorder, major depression, attention deficit hyperactivity disorder or pervasive developmental disorder.
Neuro examination	Abnormality on neurological examination (e.g., hypertonia, hypotonia, reflex asymmetry, visual field cut, nystagmus, and tics).

Our analyses focused on the first wave of data collected. MRI scans were acquired using either General Electric or Siemens 1.5 Tesla scanners. Overall, four hundred and thirty-one subjects were recruited for this project. Of this initial sample, one-hundred and fourteen subjects were excluded from our analyses (2 subject had errors in preprocessing, 10 subjects had unusable data due to motion artifacts, 41 subjects did not complete scanning, while 61 subjects were excluded due to lower resolution of their MRI scans which led to a decreased ability to localize the brain structures of interests). The demographic characteristics of the sample are displayed in Tables 2, 3, 4, 5, 6, 7, 8, along with Supplemental Tables S1 & S2. Parents of participants were asked about total household income in the last year, which includes earnings, unemployment compensation, pension or retirement income, interest, dividends, rents, social security, and all other miscellaneous sources. Incomes were then divided into 9 levels: $1–5000, $5001–10000, $10001–15000, $15001–25000, $25001–35000, $35001–50000, $50001–75000, $75001–100000,and $100001+ in the publicly available data. Parents of participants were also asked about their education and responded whether they had completed less than a 6th grade education, less than high school, graduated high school, completed some college, graduated college, obtain some graduate education, or completed graduate school.

**Table 2:** Demographic Summary for full sample (based on Wave 1 data).

Age (Average age in months for Wave 1)	126.13+/−46.59 months
Gender (Male)	207
Total n	431

**Table 3:** Demographic Summary for full sample (based on Wave 1 data).

	Father Education	Maternal Education
Less than High School	10	4
High School	86	55
Some College	116	131
College	115	144
Some Graduate Level	19	22
Graduate Level	83	73
No Information	2	2
TOTAL	431	431

**Table 4:** Demographic Summary for full sample (based on Wave 1 data).

Income at Wave 1	
<$5000	1
5001–$10,000	2
10001–15000	4
15001–25000	10
25001–35000	21
35001–50,000	82
50001–75000	104
75001–100,000	102
>100001	94
No information	11
TOTAL	431

**Table 5:** Demographic Variables for Subjects with and without MRI Scans and/or Income.

	Subjects with all variables (n = 317)	Subjects without all variables (n = 114)	
Age (Average age in months for Wave 1)	133.74+/−45.76 months	133.74+/−45.76 months	F(1,429) = 44.675, p<.001
Gender (Male)	146	61	χ^2^ = .305, p = .642

**Table 6:** Demographic Variables for Subjects with and without MRI Scans and/or Income.

Father's Education		
	Subjects with all variables (n = 317)	Subjects without all variables (n = 114)
Less than High School	7	3
High School	61	25
Some College	83	33
College	85	30
Some Graduate Level	13	6
Graduate Level	68	17
TOTAL	317	114

**Table 7:** Demographic Variables for Subjects with and without MRI Scans and/or Income.

Maternal Education		
	Subjects with all variables (n = 317)	Subjects without all variables (n = 114)
Less than High School	2	3
High School	45	25
Some College	88	33
College	107	30
Some Graduate Level	16	6
Graduate Level	59	17
TOTAL	317	114

**Table 8:** Demographic Variables for Subjects with and without MRI Scans and/or Income.

Income at Wave 1		
	Subjects with all variables (n = 317)	Subjects without all variables (n = 114)
<$5000	1	0
5001–$10,000	2	0
10001–15000	4	0
15001–25000	7	3
25001–35000	13	8
35001–50,000	53	29
50001–75000	76	28
75001–100,000	88	25
>100001	73	21
TOTAL	317	114

### Imaging Analyses

To examine the relationship between income and hippocampal gray matter, we employed a voxel-based morphometry (VBM) analytic framework with region of interest drawing. VBM is a fully automatic imaging analysis strategy which allows for the precise localization of anatomical differences between groups, involves a comparison between two groups of subjects of the local concentration of gray matter (or volume comparison) using Jacobian modulation, and has been applied to the study of various types of pathologies [21], [22], [23], [24]. The steps involved with VBM have recently been improved with the Diffeomorphic Anatomical Registration using Exponentiated Lie algebra (DARTEL) registration method [25]. Previous structural imaging research focused on the hippocampus has often employed manual segmentation protocol. Such procedures require specific anatomical expertise, are operator time consuming and may result in high intra- and inter-rater variability (as noted by [26]). Advancement in registration methods, such as DARTEL, improves the realignment of small brain structures [27], making such an analytic strategy particularly robust for quantifying the hippocampus in such a large dataset.

In this analysis, we used Statistical Parametric Mapping 8 (Wellcome Department of Cognitive Neurology: London, England) with the following steps: first, T1-weighted images were checked for scanner artifacts (e.g., extreme field inhomogeneity). Next, these volumes were segmented using custom a priori brain tissue segmentations generated by the Template-O-Matic toolbox [28]. These custom segmentations were based on the age and gender distributions of the full sample. The first author then checked the accuracy of each subjects' segmentation. If any errors were present, the bounding box or image matrix was adjusted and MRI images were reprocessed. If after this correction segments still contained errors, they were corrected by hand to remove skull, dura, and other non-brain matter.

**Figure 1:**
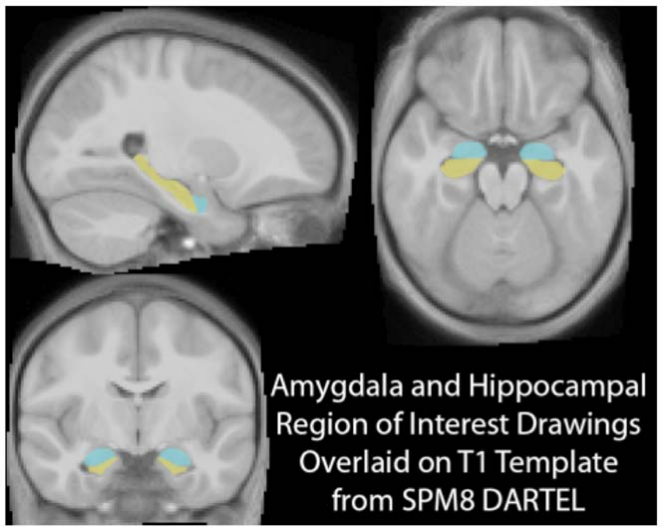
Hippocampal and amygdala region of interest drawings. The top left brain slice shows a sagittal brain slice with the hippocampus highlighted in yellow and the amygdala in turquoise, while the top right brain image shows an axial slice (with the hippocampus again highlighted in yellow and the amygdala in turquoise). The bottom left brain picture shows a coronal slice with the amygdala in turquoise and the hippocampus in yellow.

Once segmentation was completed, creation of and registration to study specific templates began. This process first involved rigidly aligning and averaging each tissue class (i.e., grey and white matter segments) for each subject. Using the initial template, an advanced non-linear registration algorithm (DARTEL) was employed to register each participant's segments to the template gray and white matter maps. The results of this registration process were then averaged to create a second template. Averaging and registering of gray and white matter segments was repeated six times. This processing pipeline allows for robust registration, while preserving the topology of the brain via constant velocity flow fields [25]. These processing procedures were recently validated as a robust approach to detecting hippocampal differences [26]. After warping the images to the final template, region of interest drawing was completed on the template through the Anatomical Automatic Labeling Toolbox [29]. The hippocampal and amygdala region of interest drawings used for our analyses are shown in Figure 1. Modulated Segments, adjusted for the non-linear registration were then generated to assess gray matter differences in relation to socioeconomic status (SES) variables.

After processing neuroimaging data from each subject with the procedures detailed above, we conducted linear regressions in Statistical Package for the Social Sciences (SPSS Inc., Chicago, IL) controlling for participant age in months, gender (dummy-coded), and whole-brain volumes entered as independent variables. The log-transformed, mid-point for each income category and the approximate number of years of education obtained by parents (<6th grade = 5 years, less than high school = 11 years, high school = 12 years, some college = 14 years, college = 16 years, some grad = 17 years and graduate level = 19 years) were also used as continuous independent variables in these analyses. Gray matter probability for the hippocampus or the amygdala (for total gray matter, as well as for the left and right side separately) was entered as the dependent variable in these regressions. The brain variables in these analyses are the unsmoothed average “modulated” gray matter density in a whole-hippocampal or amygdala region of interest drawing. Recent evaluations of registration algorithms have noted superior performance of DARTEL, with top ratings in overlap and distance measures [30]. Age, gender, whole-brain volume, and parental education were included to isolate the unique effects of income on the medial temporal lobe.

## Results

### Examining the association between income and the hippocampus

**Figure 2:**
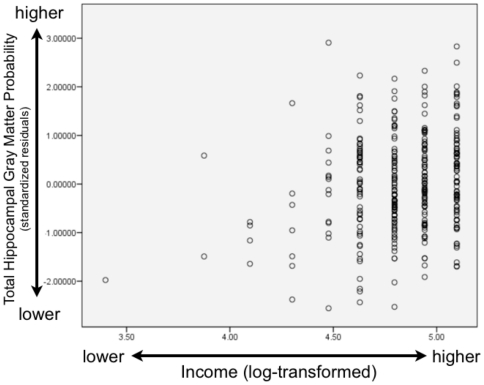
Scatterplot of Total Hippocampal Gray Matter and Income. This scatterplot shows the association between total hippocampal gray matter probability and income. Total hippocampal gray matter shown on the vertical axis is displayed as a standardized residual controlling for child's age (in months), gender (dummy-coded), and whole brain volume, while log-transformed income is displayed on the horizontal axis. Higher income is associated with greater gray matter probability.

**Figure 3:**
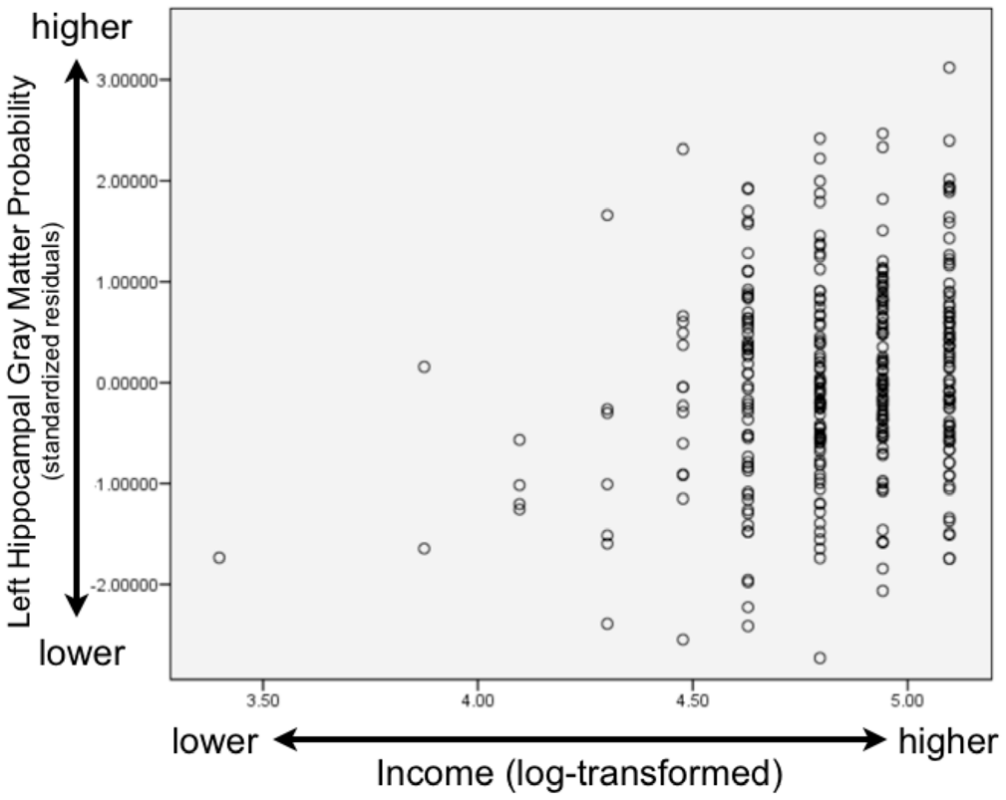
Scatterplot of Left Hippocampal Gray Matter and Income. This scatterplot shows the association between left hippocampal gray matter probability and income. Left hippocampal gray matter shown on the vertical axis is displayed as a standardized residual controlling for child's age (in months), gender (dummy-coded), and whole brain volume, while log-transformed income is displayed on the horizontal axis. Higher income is associated with greater gray matter probability.

**Figure 4:**
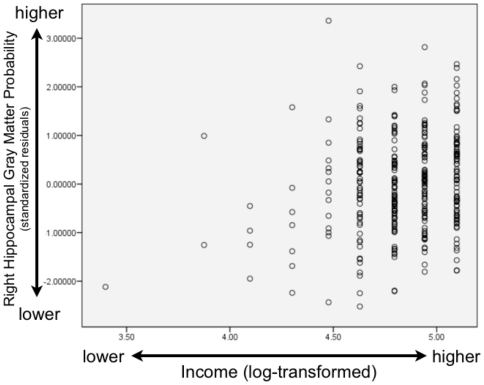
Scatterplot of Right Hippocampal Gray Matter and Income. This scatterplot shows the association between right hippocampal gray matter probability and income. Right hippocampal gray matter shown on the vertical axis is displayed as a standardized residual controlling for child's age (in months), gender (dummy-coded), and whole brain volume, while log-transformed income is displayed on the horizontal axis. Higher income is associated with greater gray matter probability.

In terms of income and the neurobiological correlates of socioeconomic status, we examined hippocampal and amygdala gray matter across a large income spectrum: participants had annual family incomes of below $5000 to above $100,000 per year. Our lowest income group is composed of families below 150% of the Federal Poverty Line (for 2010 levels, see http://aspe.hhs.gov/poverty/10poverty.shtml). As predicted, there was a relationship between income and the hippocampus, for total hippocampal gray matter (β = .145, t = 2.459, p = .014) as well as left (β = .165, t = 2.773, p = .006) and right (β = .118, t = 1.999, p = .046) hippocampal gray matter separately. Scatterplots of these associations are shown in Figure 2 (total hippocampal gray matter and income), Figure 3 (left hippocampal gray matter and income), and Figure 4 (right hippocampal gray matter and income).These results demonstrate for the first time that the hippocampus is associated with household income, as children from lower SES backgrounds had less gray matter and participants from more affluent backgrounds had greater concentrations of gray matter. All of these models included child gender entered as a dummy coded variable, child age in months, whole brain volume, parental education, and income as continous independent variables, along with the brain area of interest as the dependent variable.

To ensure specificity of these effects, we tested gray matter of the amygdala, a region adjoining the hippocampus. No such association emerged for income and amygdala gray matter (for total amygdala β = .088, t = 1.483, p = .139; for the left amygdala β = .091, t = 1.529, p = .127; for the right amygdala β = .8, t = 1.343, p = .180). The full outputs of our regression models are shown in Tables 9 & 10. Again, all of these models controlled for gender, age, whole-brain volume, and parental education. Also worthy of note, no relationship emerged between income and whole-brain volume (β = −.018, t = −.278, p = .781).

**Table 9:** Regression Output For Models Examining the Association Between the Hippocampus and Income.

Region of Interest (Dependent Variable)	Independent Variables	Unstandardized regression coefficients, Standard Error, Standardized regression coefficients, test statistics
Total Hippocampus	Maternal Education	B = −0.0001, SE = 0.003, β = −.005, t = 0.08 p = .93
	Paternal Education	B = 0.003, SE = 0.002, β = .105, t = 1.785 p = .075
	Income	B = 0.045, SE = 0.018, β = .145, t = 2.459 p = .014
Left Hippocampus	Maternal Education	B = −0.001, SE = 0.002 β = −.03, t = 0.505 p = .614
	Paternal Education	B = 0.003, SE = 0.002, β = .083, t = 1.404 p = .161
	Income	B = 0.052, SE = 0.019, β = .165, t = 2.773 p = .006
Right Hippocampus	Maternal Education	B = 0.0007, SE = 0.002, β = .02, t = −0.344, p = .73
	Paternal Education	B = 0.004, SE = 0.002, β = .122, t = 2.073 p = .039
	Income	B = 0.038, SE = 0.019, β = .118, t = 1.999 p = .046

NB: All regression models included child age (in months), gender of the child (dummy-coded), and whole-brain volume as covariates.

**Table 10:** Regression Output For Models Examining the Association Between the Amygdala and Income.

Region of Interest (Dependent Variable)	Independent Variables	Unstandardized regression coefficients, Standard Error, Standardized regression coefficients, test statistics
Total Amygdala	Maternal Education	B = −0.0003, SE = 0.002, β = −.01, t = −0.17 p = .867
	Paternal Education	B = 0.0013, SE = 0.002, β = .040, t = .679 p = .498
	Income	B = 0.031, SE = 0.021, β = .088, t = 1.483 p = .139
Left Amygdala	Maternal Education	B = −0.001, SE = 0.002, β = −.013, t = −0.22 p = .830
	Paternal Education	B = 0.001, SE = 0.002, β = .030, t = 0.509 p = .611
	Income	B = 0.034, SE = 0.022, β = .091, t = 1.529 p = .127
Right Amygdala	Maternal Education	B = −0.0002, SE = 0.002, β = −.007, t = −0.11 p = .91
	Paternal Education	B = 0.002, SE = 0.002, β = .048, t = 0.805 p = .421
	Income	B = 0.029, SE = 0.021, β = .080, t = 1.343 p = .180

NB: All regression models included child age (in months), gender of the child (dummy-coded), and whole-brain volume as covariates.

## Discussion

This study was designed to examine the possible association between household family income and the hippocampus, a brain region central to many important cognitive and emotional processes. We identified an association with the hippocampus and income, as hypothesized. The hippocampus has previously been found to be associated with quality of environmental input and stress. Taken together, these findings suggest that differences in the hippocampus, perhaps due to stress tied to growing up in poverty, might partially explain differences in long-tern memory, learning, control of neuroendocrine functions, and modulation of emotional behavior. These results are consistent with research on neuropsychological differences across the SES gradient (for review, see [31]). Farah and colleagues [13], [32] along with Rao et al. [14] found environmental stimulation and parental nurturance was related to memory functioning in childhood. Such long-term memory functions are mediated by the hippocampus [33]. Variations in hippocampal size have been associated with memory performance with larger hippocampal volumes being related to better memory performance [34]. In addition, higher levels of chronic life stress appear to be associated with smaller hippocampal volumes in adults [35]. These results add to the modest body of research examining neurobiological associations with socioeconomic status, providing one potential neurobiological mechanism through which the early environment may convey risk for a host of deleterious outcomes.

In contrast to previous research linking amygdala volume and stress [36], we did not observe associations for the amygdala and income. Amygdala quantification is very challenging and even with such a large sample size, automated methods may not be appropriate. Follow-up analyses using a different method of automated segmentation however yielded similar results (see Supplemental Materials S1). In addition, associations between the amygdala and early life stress effects may vary by age of measurement (for discussion, see [37]). For example, increases in amygdala volume may be seen early in development after the experience of stress, while small amygdala volume may occur later in development.

The structural imaging project presented here does not address issues of causation: poverty carries multiple components of environmetal risk and many factors may affect the development of brain structure. Future research should longitudinally assay both brain structure and function, as understanding both factors are likely central to truly understanding associations between neurobiological outcomes and income. Additional work should also include a variety of neuropsychological assessment, as the cognitive tests employed in this study were predominantly “prefrontal-dependent”: tapping rule acquisition and working memory. Subsequent studies must also aim to delineate the effects of household income, environmental stimulation, stress, and other variables such as possible nutritional differences related to poverty with large samples of children living in poverty. Such research designs will further increase understanding the neurobiological correlates of poverty and socioeconomic status.

This study examined a large group of children and adolescents from 5 different research sites around the United States. Although issues of race and ethnicity were not the focus of our study, these factors may be associated with variations in neural development. Preliminary analyses suggested that our effects held for Caucasian and non-Caucasian participants. Future research should focus on exploring ethnic diversity with appropriately sized samples across income categories. Of important note, the NIH data set was also designed with a plan to screenout individuals with mental health issues or very low intelligence. This design skews the sample because psychopathology and learning disorders are disproportionately represented among impoverished children. The present results therefore reflect so-called “normal” children living in poverty. This suggests that the present results likely under-represent the true effects of poverty. Alternatively one could argue that the exclusionary criteria may strengthen the implications of our results as psychopathology or learning disorders as possible explanations of the association can largely be ruled out as factors lying behind the correlation.

Understanding how environmental variations can affect neural, emotional and cognitive functioning in humans has major implications for both basic scientific questions and public policy initiatives. Such knowledge about the neural embedding of socioeconomic status, specifically poverty, may aid in the design and implementation of intervention programs addressing SES-related disparities in a cognitive and health outcomes. We found variations in socioeconomic status were associated with hippocampal volumes (as measured by gray matter probability). This finding suggests a potential neurobiological mechanism through which the early environment may convey risk for a host of deleterious outcomes from poor health to lower educational achievement. In addition to SES-related disparities, such results add to our understanding of human brain development, as we aim to further delineate how post-natal experiences may uniquely shape the brain and change behavior.

## Supporting Information

Table S1 Additional Demographic Summary for full sample (based on Wave 1 data).
(DOC)

Table S2 Demographic Variables for Subjects with and without MRI Scans and/or Income.
(DOC)

Materials S1 (DOCX)
