# Supplementary material for: Association between Income and the Hippocampus
Source: PLoS One. 2011 May 4;6(5):e18712. doi: 10.1371/journal.pone.0018712 (PMC3087752; doi:10.1371/journal.pone.0018712)
Supplement: Table S1 — Additional Demographic Summary for full sample (based on Wave 1 data). (DOC) [file pone.0018712.s001.doc]

Table S1.

Additional Demographic Summary for full sample (based on Wave 1 data)

|  | Father Race | Mother Race |
| --- | --- | --- |
| African American | 41 | 40 |
| American Indian/Alaskan Native | 2 | 1 |
| Multi-Racial | 8 | 6 |
| Asian | 9 | 8 |
| Native Hawaiian/Other Pacific Islander | 2 | 0 |
| White | 334 | 347 |
| Not Provided | 35 | 29 |

|  | Father Ethnicity | Mother Ethnicity |
| --- | --- | --- |
| Hispanic or Latino | 39 | 30 |
| Not Hispanic or Latino | 390 | 399 |
| No Information | 2 | 2 |
