# Supplementary material for: Association between Income and the Hippocampus
Source: PLoS One. 2011 May 4;6(5):e18712. doi: 10.1371/journal.pone.0018712 (PMC3087752; doi:10.1371/journal.pone.0018712)
Supplement: Table S2 — Demographic Variables for Subjects with and without MRI Scans and/or Income. (DOC) [file pone.0018712.s002.doc]

Table S2.

Demographic Variables for Subjects with & without MRI Scans and/or Income

| Father Race | | |
| --- | --- | --- |
|  | Subjects with all variables (n=317) | Subjects without all variables (n=114) |
| African American | 30 | 11 |
| American Indian/Alaskan Native | 2 | 0 |
| Multi-Racial | 8 | 0 |
| Asian | 6 | 3 |
| Native Hawaiian/Other Pacific Islander | 2 | 0 |
| White | 245 | 89 |
| Not Provided | 24 | 11 |

| Mother Race | | |
| --- | --- | --- |
|  | Subjects with all variables (n=317) | Subjects without all variables (n=114) |
| African American | 31 | 9 |
| American Indian/Alaskan Native | 1 | 0 |
| Multi-Racial | 3 | 3 |
| Asian | 4 | 4 |
| Native Hawaiian/Other Pacific Islander | 0 | 0 |
| White | 256 | 91 |
| Not Provided | 22 | 7 |

| Father Ethnicity | | |
| --- | --- | --- |
|  | Subjects with all variables (n=317) | Subjects without all variables (n=114) |
| Hispanic or Latino | 28 | 11 |
| Not Hispanic or Latino | 287 | 103 |
| No Information | 2 | 0 |

| Mother Ethnicity | | |
| --- | --- | --- |
|  | Subjects with all variables (n=317) | Subjects without all variables (n=114) |
| Hispanic or Latino | 23 | 7 |
| Not Hispanic or Latino | 292 | 107 |
| No Information | 2 | 0 |
