# Supplementary material for: Association between Income and the Hippocampus
Source: PLoS One. 2011 May 4;6(5):e18712. doi: 10.1371/journal.pone.0018712 (PMC3087752; doi:10.1371/journal.pone.0018712)
Supplement: Materials S1 — (DOCX) [file pone.0018712.s003.docx]

Supplemental analyses

In addition to voxel-based morphometry analyses with region of interest drawings, amygdala and hippocampal volume was quantified by registering a parcellated brain (Davatzikos, Genc, Xu, & Resnick, 2001) via diffeomorphic warping to each individual subject. The warping algorithm employed, Symmetric Normalization (Avants & Gee, 2004) was recently judged as one of the best available in a comparison of 14 non-linear registration routines (Klein et al., 2009).

In regression models equivalent to those detailed in the main manuscript, we find a similar association between income and the hippocampus (total hippocampal volume β=.104, p=.021; left hippocampal volume β=.107, p=.018; right hippocampal volume β=.092, p=.048). The association between income and amygdala, much like those reports in the main manuscript, were non-significant (total amygdala volume β=.013, p=.763; left amygdala volume β=.037, p=.402; right amygdala volume β=-,013 p=.773).

References

Avants B, Gee JC (2004) Geodesic estimation for large deformation ana- tomical shape averaging and interpolation. Neuroimage 23:S139 –S150.

Davatzikos C, Genc A, Xu D, & Resnick SM. (2001). Voxel-Based Morphometry Using the RAVENS Maps: Methods and Validation Using Simulated Longitudinal Atrophy NeuroImage 14: 1361-1369.

Klein A, Andersson J, Ardekani BA, Ashburner J, Avants B, Chiang MC, Christensen GE, Collins DL, Gee J, Hellier P, Song JH, Jenkinson M, Lepage C, Rueckert D, Thompson P, Vercauteren T, Woods RP, Mann JJ, Parsey RV (2009) Evaluation of 14 nonlinear deformation algorithms applied to human brain MRI registration. Neuroimage 46:786 – 802.
